# Biodiversity loss disrupts seasonal carbon dynamics in a species‐rich temperate grassland

**DOI:** 10.1002/ecy.70091

**Published:** 2025-05-08

**Authors:** Jules Segrestin, Aleš Lisner, Lars Götzenberger, Tomáš Hájek, Eva Janíková, Veronika Jílková, Marie Konečná, Tereza Švancárová, Jan Lepš

**Affiliations:** ^1^ Department of Botany Faculty of Science, University of South Bohemia České Budějovice Czech Republic; ^2^ Institute of Botany of the Czech Academy of Sciences Třeboň Czech Republic; ^3^ Department of Experimental Plant Biology Faculty of Science, University of South Bohemia České Budějovice Czech Republic; ^4^ Biology Centre of the Czech Academy of Sciences Institute of Soil Biology and Biogeochemistry České Budějovice Czech Republic

**Keywords:** aboveground biomass, belowground biomass, biodiversity loss, dominant species, ecosystem functioning, litter decomposition, net ecosystem C exchange, phenological complementarity, seasonal carbon dynamics, soil organic carbon

## Abstract

Biodiversity loss poses a significant threat to ecosystem functioning. However, much of the empirical evidence for these effects is based on artificial experiments that often fail to simulate the structure of natural communities. Hence, it is still unclear whether natural diversity losses would significantly affect the functioning of “real‐world” ecosystems. As subordinate and rare species constitute most of the diversity in natural communities and are often more vulnerable to local extinction, we evaluated their contribution to ecosystem functioning in a naturally species‐rich grassland. We focused on two mechanisms by which they can support ecosystem functions: redundancy and complementarity. We conducted two long‐term field experiments (>6 years) simulating contrasting biodiversity loss scenarios through the manual removal of plant species and measured the consequences of species loss on various ecosystem functions related to carbon dynamics. The latter were examined seasonally to explore diversity effects outside the typical peak of vegetation. We found that dominant removal led to substantial reductions in aboveground phytomass and litter production and altered the annual carbon fixation capacity of the vegetation, highlighting the pivotal role of dominant species in driving ecosystem functioning. Despite high species diversity, other species could not fully compensate for the loss of a single dominant even after more than 25 years, challenging assumptions about redundancy. Complementarity effects were not detected at the peak of vegetation but were evident in early spring and autumn when subordinate and rare species enhanced ecosystem functions. Surprisingly, belowground phytomass, soil organic carbon content, and litter decomposition were unaffected by species removal, suggesting complex interactions in belowground processes. These findings underscore the importance of dominant species in maintaining ecosystem functioning and emphasize the need for nuanced approaches to studying biodiversity loss in real‐world communities. Comprehensive seasonal measurements are essential for accurately discerning the effects of biodiversity on ecosystem dynamics and informing effective conservation strategies that maintain ecosystem functioning.

## INTRODUCTION

In recent decades, there has been growing recognition of the critical role biodiversity plays in shaping ecosystem functions and services (e.g., Cardinale et al., 2012; Isbell et al., 2011; Loreau et al., 2001; Tilman et al., 2014). Yet, most of the empirical evidence revealing the beneficial effect of biodiversity on ecosystem functioning relies on artificial experiments (often referred as BEF experiments) in which communities of varying diversity are randomly assembled (e.g., Hector et al., 1999; Tilman et al., 2001; Weisser et al., 2017). Although these experiments have been proven successful in demonstrating the importance of biodiversity in multiple ecosystem functions (e.g., Cardinale et al., 2007; Chen et al., 2019; Ravenek et al., 2014), results from field observations are often less conclusive (Freitag et al., 2023; Genung et al., 2020; Hagan et al., 2021; van der Plas, 2019). While some authors argue that traditional BEF experiments can provide realistic results (Duffy, 2009; Jochum et al., 2020), it is still unclear whether the conclusions from these experiments can help us predict the effects of biodiversity loss on “real‐world” ecosystems (Lepš, 2004; Wardle, 2016).

An alternative approach to traditional BEF experiments has been proposed to study the contribution of species to ecosystem functions. It consists of the long‐term repeated removal of plant species from natural communities (see Sasaki et al., 2017; Smith & Knapp, 2003) instead of establishing artificial, and often randomly assembled, communities. Results from removal experiments are often considered useful for understanding the ecosystem effects of local, nonrandom extinctions, changes in the natural abundance of species, and complex interspecific interactions (Díaz et al., 2003). Moreover, unlike synthetic assemblage experiments, removal experiments have the advantage of using naturally assembled communities, incorporating natural biotic and abiotic filtering and realistic distributions of species abundances (Munson & Lauenroth, 2009). Overall, removal experiments promise a deeper understanding of the consequences of biodiversity loss in natural ecosystems. Yet, this approach has remained poorly adopted (but see Lisner et al., 2023; Sasaki et al., 2017).

Understanding the consequences of “real‐world” diversity loss requires elucidating the role of low‐abundance species in ecosystem functions (Dee et al., 2019; Lyons et al., 2005; Mariotte, 2014). Indeed, these species constitute most of the diversity in natural communities, which typically comprise a few dominant species and numerous subordinate and rare species. Additionally, due to their low abundance, these species are often more vulnerable to local extinction, particularly in the context of ongoing declines in diversity (Genung et al., 2020; Smith & Knapp, 2003). Although the contribution of species of low abundance in ecosystem functioning has often been considered negligible (in line with the mass‐ratio hypothesis: Grime, 1998), Lyons et al. (2005) proposed three mechanisms through which these species can have a significant contribution to ecosystem functions that relate to the concepts of (1) redundancy, (2) complementarity (also referred as aggregated effect of diversity), and (3) key(stone) species.

Redundancy is defined as the ability of species to sustain ecosystem functioning despite biotic or abiotic hazards affecting specifically dominants. It is often assumed that a high species diversity can ensure ecosystem functioning by compensating for the drop of function associated with a strong decrease or even loss of a high‐performing species (Walker et al., 1999; Yachi & Loreau, 1999). Complementarity, on the other hand, is defined as the ability of species to enhance ecosystem functioning beyond what is achievable with dominant species alone, often through niche partitioning that enhances resource use efficiency and productivity (Barry et al., 2019). Finally, key species represent species of low abundance having a disproportionate effect on an ecosystem function through their unique characteristics (see examples in Lyons et al., 2005). Since the identification of key species often necessitates complex experimental designs—as their effects are often function‐ and ecosystem‐specific—we decided to focus on the first two mechanisms in this study.

We propose that the removal of species from naturally assembled communities can help us to evaluate the redundancy and complementarity provided by subordinate and rare species. By experimentally removing the most dominant species and examining the long‐term consequences for ecosystem functioning, we can test whether the diversity of the remaining plant species provides enough redundancy to fully compensate for the loss of a dominant. Additionally, complementarity can be evaluated through different levels of removal of subordinate and rare species from the community, keeping the most abundant species. A consistent decrease in ecosystem functioning with the removal of low‐abundance species would indicate ecosystem functions that are not fully supported by dominants and demonstrate the complementary role of low‐abundance species to ecosystem functioning.

The vast majority of studies testing the effects of diversity on ecosystem functions have focused on aboveground productivity and its temporal stability (e.g., Hector et al., 1999, 2010; Tilman et al., 2014). Although other studies have tested diversity effects on other functions such as litter decomposition (Guo et al., 2020; Kazakou et al., 2022), soil carbon sequestration (Chen et al., 2018, 2020), or nutrient cycling (Hooper & Vitousek, 1998), studies reporting simultaneous effects on various ecosystem functions (often referred as multifunctionality) are still comparatively scarce (but see Dietrich et al., 2024; Dooley et al., 2015; Hector & Bagchi, 2007; Maestre et al., 2012; Valencia et al., 2015). Hence, there is a need for studies investigating the role of diversity across different compartments of the ecosystem simultaneously. As an important addition, we propose investigating the role of diversity across seasons. This approach could help us to deepen our understanding of the role of plant diversity in the functioning of ecosystems by adding a temporal aspect to our theoretical expectations. More specifically, it could be hypothesized that the complementarity effect of subordinate and rare species to ecosystem functions may be more pronounced in the early and late seasons, outside of the dominant‐driven peak of the vegetation. Surprisingly, this idea has been rarely tested as most previous studies analyzed data collected once a year, usually at the peak of the growing season.

**FIGURE 1:**
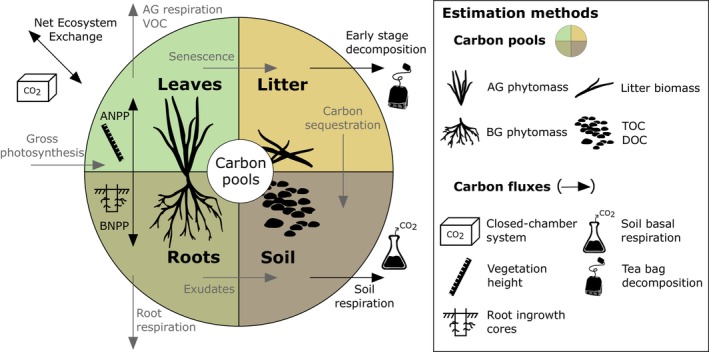
Main pools (colored areas) and fluxes (black and gray arrows) of carbon in temperate grasslands. Four major pools were identified in the system and are interconnected by different fluxes. We used various estimation methods to determine the qualitative effect of species loss treatments on the four carbon pools and on a selection of carbon fluxes (black arrows only). For practical reasons, the gross photosynthesis, and the ecosystem respiration (including the respiration of autotrophs and heterotrophs) could not be measured seasonally. However, we monitored the net ecosystem CO_2_ exchange, corresponding to the net difference between the latter. Note that this simplified representation of carbon dynamics focuses on the primary production and soil microorganisms and ignores higher trophic levels which constitute other carbon pools interacting through various fluxes (e.g., herbivory, respiration, soil carbon sequestration). A·B·NPP, above·below·ground net primary productivity; AG, aboveground; BG, belowground; DOC, soil dissolved organic carbon; TOC, soil total organic carbon, VOC, volatile organic compounds. Illustration credit to Jules Segrestin and Aleš Lisner.

Here, we present results from two long‐term removal experiments conducted in a species‐rich temperate grassland. Temperate meadows are among the most threatened hotspots of biodiversity (Habel et al., 2013) and it is imperative to assess the consequences of species loss on its functioning. We investigated the consequences of two removal scenarios (i.e., removal of a dominant and removal of low‐abundance species) on the seasonal carbon (C) dynamics (Figure 1). This approach appears particularly relevant since grasslands store approximately one third of global terrestrial C stocks (Bai & Cotrufo, 2022) and exhibit a remarkable capacity for soil C sequestration, which stems from intricate interactions among C pools and fluxes, notably in belowground compartments (see Figure 1). Moreover, studying C dynamics allows a comprehensive view of the simultaneous effects of species loss on various compartments of the ecosystem. To our best knowledge, our study represents the first attempt to investigate the impact of a realistic diversity loss on the main ecosystem C pools and fluxes at such a detailed temporal resolution and promises to deepen our understanding of the underlying mechanisms.

Overall, we addressed the following questions:

1. How did the long‐term removal of one single dominant species affect the C dynamics of the studied meadow? Did the numerous remaining species include redundant species capable of fully compensating for this loss?
2. Does the loss of subordinate and rare species disrupt C pools and seasonal fluxes? Accordingly, can we find evidence for species complementarity in this highly diverse ecosystem?
3. Are the effects of species loss on these ecosystem functions consistent across seasons, or do they vary seasonally depending on the removal scenario?

## MATERIALS AND METHODS

### Study site and experimental designs

The study was conducted on an oligotrophic wet meadow located 10 km southeast of České Budějovice, Czech Republic (48°570 N, 14°360 E, altitude 510 m). The site has traditionally been extensively mown for centuries. The vegetation is species‐rich with an average species richness of *c*. 30 species per 0.25 m^2^ and dominated by grasses, especially *Molinia caerulea* (L.) Moench. The latter represents *c*. 30% of the total aboveground biomass on average, and no other species can be identified as a true codominant.

We used two neighboring manipulative experiments to investigate the effects of the long‐term removal of plant species on C pools and fluxes.

In the first experiment (DomRem, hereafter), the most dominant species, *M. caerulea*, was manually weeded in 2 m × 2 m plots since 1995, and new individuals growing afterward were removed annually. After a few years, only a few individuals had to be removed occasionally. We collected data on 12 plots (six controls and six treated plots). Treatments were randomized in three blocks. None of the plots have been fertilized, but they received different treatments of mowing in the past. Since 2016, all plots have been mown once a year at the peak of the vegetation (i.e., the traditional management of the site). More details on the experimental design are given in Lepš et al. (2018) and references therein.

In the second experiment (SubRarRem, hereafter), subordinate and rare species were weeded in 1 m × 1 m plots since 2016. In each plot, all species abundances were determined in 2016 at the peak of the vegetation. Four levels of species removal were applied, keeping the 12, 6, 3, or 1 most abundant species. The treatments were maintained twice a year—once in early spring and once in late summer—by removing resprouting individuals, creeping stems from the surrounding vegetation, and new seedlings. To disentangle the effects of species loss from that of the disturbance induced by the weeding, we established disturbed controls where plant individuals were weeded irrespective of the species identity. The biomass removed in these plots corresponded to the average biomass removed on the same date in other removal treatments. Control plots (intact controls and disturbed controls) and treated plots (four levels of species removal) were replicated five times and organized in a 30 plots Latin‐square‐like design. Each row of the experiment contained all treatments and constituted an independent block. More details on the experimental design are given in Lisner et al. (2023).

### Data collection: C pools

The aboveground phytomass was harvested at the peak of the vegetation (14 and 21 June 2022 for the DomRem and SubRarRem experiments, respectively), in the central 0.5 m × 0.5 m area of each plot. The harvest was done concurrently with the single annual mowing of the experiments, which is part of the traditional meadow management. Hence, the biomass collection does not represent an additional disturbance for the studied system. Living parts of vascular plants were sorted (removing mosses and litter) and oven‐dried at 80°C for 48 h.

The litter biomass was collected at the end of the vegetative season when most of the aboveground phytomass was senescent. In each plot, we harvested all standing litter in four marked 0.1 m × 0.1 m areas twice (21 November 2022 and 4 January 2023 in both experiments). All samples were oven‐dried at 60°C for 48 h, and the plot litter biomass corresponds to the sum of both successive samplings.

The belowground phytomass was harvested at the date of mowing by collecting one soil‐core of 5 cm in diameter and 40 cm deep in each plot. Samples were collected at least 20 cm from the plot edges to reduce the presence of roots from the plants growing in the surrounding plots and the nearby vegetation. All cores were washed over a sieve of mesh size 0.5 mm with clear water, and living plant biomass was manually sorted into roots and other belowground organs such as bulbs and rhizomes. The few roots of trees growing near the experiment could be identified and were excluded from the samples. All the samples were oven‐dried at 60°C for 48 h.

The content of soil organic C was determined from one soil core in each plot of 1.5 cm in diameter and 20 cm deep, collected at the date of mowing. Each soil sample was carefully sieved through a 2‐mm mesh to remove plant material and small rocks. The total organic C (TOC) was determined in air‐dried and ball‐milled samples, using a TOC‐LCPH/CPN model TOC analyzer coupled with an SSM‐5000A solid sample module (Shimadzu). Dissolved organic C (DOC) was extracted in deionized water (dH_2_O) (1:10 sample:dH_2_O ratio) and analyzed using a TOC‐LCPH/CPN analyzer (Shimadzu).

### Data collection: Seasonal C fluxes

The net ecosystem CO_2_ exchange (NEE) was determined using a closed‐chamber system. We used a 1 m × 1 m × 0.5 m transparent chamber equipped with two electric fans for air mixing and connected to a portable gas analyzer (LI‐6400, Licor, USA). The CO_2_ air concentration in the chamber was recorded every second for 1 min, immediately after placing the chamber on the plot. A metallic frame placed on the ground provided the base for the chamber, and towels were used to prevent air leaks. NEE was calculated as the rate of C exchange between the ecosystem and the atmosphere (expressed in milligrams of carbon dioxide per square meter per second) by selecting a period of at least 30 s characterized by a stable linear change in CO_2_ air concentration within the chamber. NEE measurements were done once a month from February to November 2022, occurring between 9 and 12 AM on a sunny day to coincide with peak photosynthetic activity of the plant communities. Therefore, our measurements do not correspond to a daily C budget. In June, NEE was monitored twice, before and after the mowing of each experiment.

Seasonal changes in aboveground phytomass were determined by repeated measurements of the vegetation height (see Appendix S1: Figure S1). We measured the height of the uppermost green vegetative tissue in five marked locations per plot (four corners and center of the central 0.5 m × 0.5 m area) from February to December 2022, at a frequency of *c*. 10 days. The vegetation height of each plot corresponded to the mean value of these five measurements.

The seasonal root growth was determined using small ingrowth cores that consisted of plastic mesh cylinders of 3 cm in diameter, 15 cm in height, and 5 mm in mesh size. Three ingrowth cores were installed on 6 December 2021, on three edges of the inner 0.5 m × 0.5 m area of each plot. Ingrowth cores were harvested and replaced after each season (17 March 2022, 21 June 2022, 29 September 2022, and 4 January 2023). At each installation date, they were filled with fresh soil collected in autumn 2021, from the same site, near the experiments. The soil was collected from the top 15 cm and sieved through a 5 mm mesh to remove gravel, stones, and plant material, homogenized, and oven‐dried at 40°C for several days. We harvested ingrowth cores by carefully cutting all roots around the mesh with a knife before pulling the cylinder out of the ground. After harvest, ingrowth cores were washed with clear water, and we hand‐sorted the belowground phytomass (mainly roots). A few roots of trees growing near the experiment could be identified and were excluded from the samples. Root samples were oven‐dried at 60°C for 48 h. The seasonal root growth per plot corresponded to the dry mass of roots produced in the three ingrowth cores expressed in kilograms per square meter of ground area.

Basal soil respiration corresponds to the respiration rate of soil microorganisms in standardized conditions. Soil cores of 2 cm in diameter and 20 cm deep were collected in each plot for each season (Winter: 15 February, Spring: 25 May, Summer: 2 August, and Autumn: 14 November 2022). Each soil sample was carefully sieved through a 2‐mm mesh to remove plant material and gravel and stored at 4°C overnight. Soil samples of 10 g were incubated for 24 h in sealed 100‐mL glass vessels. We used a syringe to collect a 10‐mL gas sample through the rubber lid and stored it in a 3‐mL pre‐evacuated vial (Exetainer, Labco Ltd., UK). Gas samples were analyzed within 24 h with an HP 5890 gas chromatograph. We are fully aware of the disturbance caused to the soil samples by sieving and weighing into the glass vessels. Nevertheless, we considered the disturbance effect similar in all the samples and decided to incubate the soil samples without preincubation, which would unnecessarily prolong the sample analyses.

The seasonal rates of early‐stage litter decomposition were determined using the tea bag methodology (Keuskamp et al., 2013). We used two tea types with contrasting decomposability: a fast‐decomposing green tea and a slow decomposing rooibos tea (the exact product references can be found in Keuskamp et al., 2013). Following the standardized protocol, we buried two bags of each tea type in each plot at a depth of *c*. 8 cm and retrieved them after *c*. 3 months. This procedure was repeated for each season, using the same positions in the plots (i.e., along the four edges of the 0.5 m × 0.5 m area of each plot). The litter mass loss was determined by comparing the dry mass of remaining tea with its initial mass before decomposition. The litter mass loss of each tea type in each plot corresponded to the mean value of the two replicates.

### Statistical analyses

All statistical analyses were conducted on R version 4.4.0 (R Core Team, 2020). We ran linear mixed‐effects models (“lme4” package: Bates et al., 2015) and Wald χ^2^ tests (“car” package: Fox & Weisberg, 2018) to test the effect of species removal (the experimental treatment) on each C pool and flux. Both experiments were analyzed separately and had their own set of control plots. In both experiments, the level of species removal was treated as a categorical variable (control vs. removal of *M. caerulea* in the DomRem experiment, and five diversity levels in the SubRarRem experiment). Post hoc Tukey's tests were used to identify differences between individual diversity levels in the second experiment (“multcomp” package: Hothorn et al., 2008). In the SubRarRem experiment, we were also interested in general trends rather than testing whether each diversity level differed from the others. Therefore, we ran models where the treatment was treated as a continuous variable, using the log‐transformed targeted species richness (i.e., 1, 3, 6, 12 for the corresponding removal treatments and 26 species for control plots corresponding to the observed mean species richness). All models included the block as a random factor to account for the small variations due to spatial heterogeneity in the experiments. For the SubRarRem experiment, we had intact control plots and disturbed control plots. We compared models using either type of control, and the results remained qualitatively similar, suggesting a negligible effect of the disturbance induced by the species removal. Consequently, we present only results using intact plots.

All C pools (aboveground and belowground phytomass, litter biomass, TOC, and DOC) were log‐transformed before statistical analyses to improve normality and avoid heteroscedasticity.

For the repeated measurements of C fluxes, we first ran a linear mixed‐effects model akin to a repeated measures ANOVA, including the treatment, the sampling date, and their interaction as fixed effects. The model also included the plot and block identities as random factors. When the treatment or the interaction factor was significant (*p* < 0.05), we ran independent models for each sampling date to identify specific responses. As per the high frequency of NEE and vegetative height measurements, these multiple individual tests should be interpreted carefully due to the temporal autocorrelation between successive sampling and the increasing probability of type I error with multiple testing. Therefore, only consistent and significant trends across successive measurements were interpreted as evidence of an effect of the removal treatments. Other C fluxes were less subject to temporal autocorrelation as they were sampled either once per season (for the basal soil respiration) or corresponded to a cumulative process over the whole season (for root growth and litter mass loss). Therefore, individual tests could be interpreted with more confidence. Vegetative height and root growth were log‐transformed before statistical analyses to improve normality and avoid heteroscedasticity.

## RESULTS

### Effect of species removal on important C pools

**FIGURE 2:**
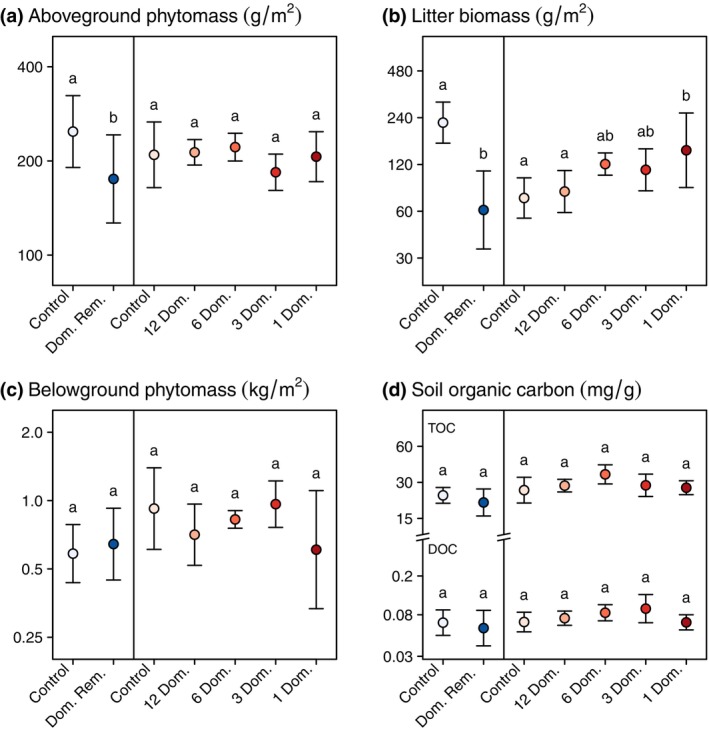
Effect of the long‐term removal of plant species on major ecosystem carbon pools: (a) aboveground phytomass at the peak of the vegetation, (b) aboveground litter biomass at the end of the vegetative season, (c) belowground phytomass, and (d) total (TOC) and dissolved (DOC) soil organic carbon. All variables are represented on a log‐transformed axis which is broken for the soil organic carbon (panel d) for readability. In each panel, the left part represents results from a removal experiment where only the most abundant species, *Molinia caerulea*, is removed, and the right part (red dots) represents results from a removal experiment where subordinate and rare species were removed, keeping only 12, 6, 3, or the 1 most abundant species. Points are mean values of replicated plots and error bars represent SDs. The statistical values of mixed models (Wald χ^2^ tests) are given in Table 1 and points that share the same letter are not significantly different: post hoc Tukey test, *p* > 0.05.

In DomRem, the long‐term removal of the one most dominant species, *M. caerulea*, resulted in a *c*. 30% decrease in the aboveground phytomass at the peak of the vegetation (Figure 2a, Table 1) and none of the remaining species were able to fully compensate for this species loss. On the contrary, we found no effect of the removal of subordinate and rare species on the aboveground phytomass in SubRarRem. Therefore, monocultures of the most dominant species produced as much aboveground phytomass as communities composed of more than 20 species (Figure 2a, Table 1). The litter biomass, collected at the end of the vegetation season, was tightly related to the removal treatments (Figure 2b, Table 1). In DomRem, the removal of *M. caerulea* reduced the litter production by *c*. 73% (Figure 2b, Table 1). On the other hand, the removal of subordinate and rare species in SubRarRem resulted in a significant increase in litter production, and monocultures had a 2‐fold increase in the litter biomass compared with controls (Figure 2b, Table 1, Appendix S1: Table S1), likely due to the increase in abundance of *M. caerulea* in response to species removal. In both experiments, we found no significant effect of removal treatments on the belowground phytomass (Figure 2c, Table 1). Similarly, we found no significant effect of removal treatments on TOC or DOC (Figure 2d, Table 1).

**TABLE 1:** Effects of the long‐term removal of plant species on major ecosystem carbon pools.

	Aboveground biomass	Belowground biomass	Litter	Total soil organic carbon	Dissolved soil organic carbon
DomRem					
χ^2^	4.4	0.3	29.9	1.2	0.4
df	1	1	1	1	1
*p* value	**0.035**	0.558	**<0.0001**	0.265	0.523
SubRarRem					
χ^2^	3.7	5.4	13.9	8.7	8
df	4	4	4	4	4
*p* value	0.444	0.248	**0.008**	0.069	0.093

*Note*: Statistical values of Wald χ^2^ tests on linear mixed‐effects models including the removal treatment as a fixed effect and the block identity as a random factor. DomRem is the experiment where the dominant species (*Molinia caerulea*) was removed from the vegetation and SubRarRem is the experiment where 5 levels of subordinate and rare species removal were conducted. Significant effects (*p* values < 0.05) are shown in bold.

### Effect of species removal on seasonal C fluxes

**FIGURE 3:**
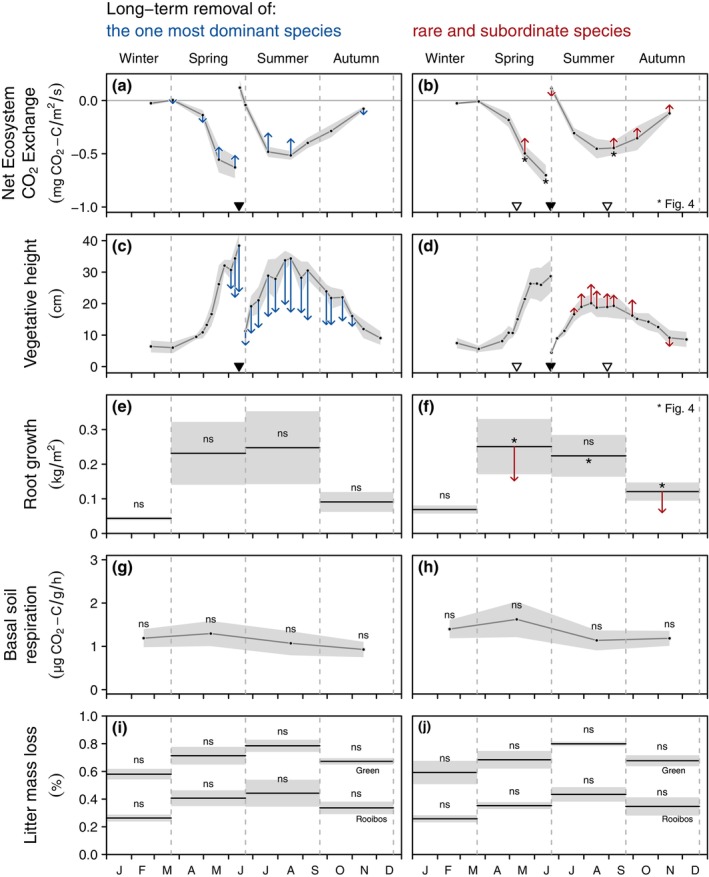
Effects of the long‐term removal of plant species on the seasonal dynamics of selected ecosystem carbon fluxes. Black dots represent the mean values measured in control plots at a given sampling date (panels a, b, c, d, g, and h) while horizontal black lines represent the mean values over the whole season (panels e, f, i, and j). Gray areas represent SDs of controls. When significant (*p* < 0.05, see Appendix S1: Table S2), the effect of species removal is represented by a colored arrow at the corresponding sampling date or season. Arrows point to the mean value of the plots where *Molinia caerulea* was removed (left panels), or to the mean value of 1‐species plots in the experiment where subordinate and rare species were removed (right panels). Both experiments were mown at the peak of the vegetation (black triangles) and the experiment removing subordinate and rare species was manually weeded twice a year (open triangles). Selected relationships identified by an asterisk are shown in detail on Figure 4.

**FIGURE 4:**
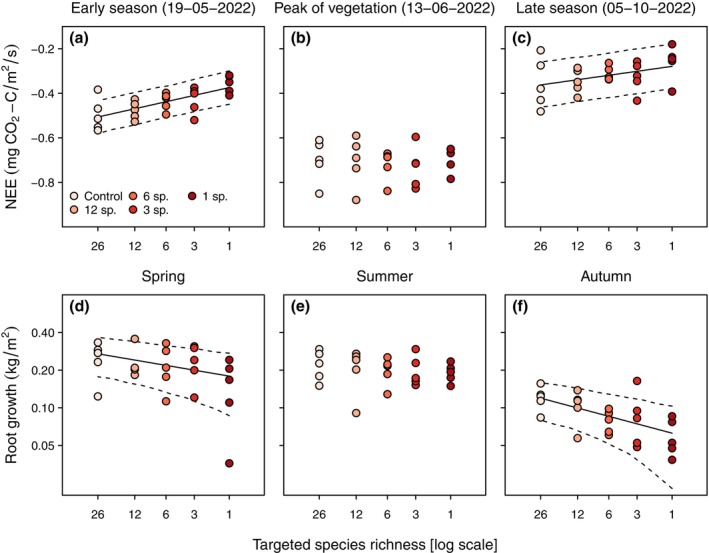
Selection of sampling dates (identified by an asterisk on Figure 3) showing the effect of the long‐term removal of subordinate and rare species on the (a, b, c) net ecosystem CO_2_ exchange (NEE) and (d, e, f) growth of belowground phytomass in ingrowth cores. The removal of subordinate and rare species reduced the ecosystem C fixation (increasing NEE) only in the early and late seasons and reduced the root production only in spring and autumn. Each dot represents a plot value (*n* = 5 for each treatment). When significant, a black line represents the regression line of the linear mixed‐effects model, and dashed lines delineate the CI. Statistical values are given in Table 2.

In both experiments, the seasonal monitoring of NEE showed an increasing rate of ecosystem C fixation (negative values of NEE) in spring, concurrent with the development of the vegetation (Figure 3a,b). In June, the mowing of both experiments resulted in a short period of C efflux (positive values of NEE), followed by an increasing rate of ecosystem C fixation in summer with the regrowth of the vegetation (Figure 3a,b). From August, the rate of C fixation decreased until the end of the year (Figure 3a,b) but NEE remained negative. In both experiments, species removal resulted in a decrease in the rate of C fixation (see Appendix S1: Table S2), but this effect occurred at different times. In DomRem, the removal of *M. caerulea* strongly reduced the ecosystem C fixation rate in late spring and summer but had little effect on NEE in early spring and autumn (Figure 3a, Appendix S1: Table S3). Conversely, the removal of subordinate and rare species in SubRarRem had little effect on the NEE during the main vegetative season (late spring and summer) but reduced the rate of C fixation in mid spring, late summer, and autumn (Figures 3b and 4a–c, Table 2, Appendix S1: Table S3).

**TABLE 2:** Effects of the removal of subordinate and rare species on NEE and root growth (log‐transformed) at different seasons.

	Early season	Peak of vegetation	Late season
NEE	19‐05‐2022	13‐06‐2022	05‐10‐2022
χ^2^	20.4	0.01	4.7
df	1	1	1
*p* value	**<0.0001**	0.899	**0.030**
Root growth	Spring	Summer	Autumn
χ^2^	7.7	1.6	17.8
df	1	1	1
*p* value	**0.006**	0.207	**<0.0001**

*Note*: Statistical values of Wald χ^2^ tests on linear mixed‐effects models including the removal treatment as a fixed effect and the block identity as a random factor. In these models, the 5 levels of targeted species richness were treated as a continuous variable to test general trends rather than testing whether each diversity level differed from the others. Significant effects (*p* values <0.05) are shown in bold.

The vegetation height followed an opposite trend compared with that of NEE: an exponential increase in spring until the peak of the vegetation in June, a second growth period after the mowing of the vegetation and until August, and a steady decrease due to the vegetation senescence in autumn (Figure 3c,d). In both experiments, species removal resulted in significant changes in the vegetation height (see Appendix S1: Table S2). In DomRem, *M. caerulea* removal significantly decreased the vegetation height at the peak of the vegetation in June and consistently reduced the vegetation height during the second growth period (Figure 3c, Appendix S1: Table S4). On the other hand, the removal of subordinate and rare species in SubRarRem did not affect the vegetation height during the first growth period (before mowing) but increased height at the second peak of vegetation in August (Figure 3d, Appendix S1: Table S4).

Root growth was slow in winter, increased in spring and summer, and decreased again in autumn (Figure 3e,f). We found no effect of the removal of *M. caerulea* on the root production in either season in DomRem (Figure 3e, Appendix S1: Table S2). However, the removal of subordinate and rare species significantly affected root growth in SubRarRem (Appendix S1: Table S2). More specifically, separate analyses for each season showed that the removal of subordinate and rare species reduced root production in spring and autumn (Figure 3f). Monocultures produced *c*. 40% less belowground phytomass than controls in spring and *c*. 50% less in autumn. However, no effect was found in winter and summer (Figure 3f, Appendix S1: Table S5). The low plant activity in winter can explain the lack of response to species removal during this season. More interestingly, we found that monocultures produced as much root biomass as controls in summer and one single dominant species fully compensated for the loss of more than 20 species (Figures 3f and 4d–f, Table 2).

Seasonal measurements of soil basal respiration (Figure 3g,h) and litter mass loss (Figure 3i,j) were not affected by the removal of species in either experiment (Appendix S1: Table S2).

## DISCUSSION

Our study aimed to investigate the contribution of subordinate and rare species to seasonal C dynamics at the ecosystem level. Indeed, elucidating their importance for ecosystem functioning is imperative to better predict the functional consequences of real‐world biodiversity loss at a local scale. Two removal experiments were conducted to evaluate the redundancy (ability to fully compensate for the loss of a dominant) and complementarity (enhanced functioning compared with communities composed of dominants only) provided by subordinate and rare species in a species‐rich temperate grassland. We did not aim at producing a full C budget for the studied meadow. Instead, we investigated the effects of both scenarios of species loss on selected proxies of C pools and seasonal fluxes (also proposed in Catovsky et al., 2002).

### Biodiversity as a source of redundancy?

It is often assumed that high species diversity allows functional redundancy between species which can act as insurance for maintaining ecosystem functions against abiotic and biotic hazards (Walker et al., 1999; Yachi & Loreau, 1999). However, our findings reveal that even an exceptionally high plant diversity (more than 20 species per 0.25 m^2^), including a large taxonomic and functional diversity, could not fully compensate for the loss of a single dominant species. Surprisingly, even after more than 25 years of experimental treatment, no species has emerged to fill the role of dominant, either within the local or regional species pool. Specifically, the long‐term removal of *M. caerulea* from the species‐rich grassland community resulted in a 30% decrease in aboveground phytomass at the peak of vegetation (see comparable results in Pinder, 1975; Smith et al., 2020; Smith & Knapp, 2003), consistently reduced vegetation height during both growing periods, and significantly diminished the aboveground litter production. Over the year, the long‐term removal of *M. caerulea* resulted in a significant decrease in the C fixation capacity of the plant community, likely reducing the annual input of C at the ecosystem level. Pan et al. (2016) also reported a strong decline in the ecosystem C fixation when removing dominant plant functional groups. While the scenario of permanently losing a dominant species may seem unlikely without a complete shift in species composition, temporary losses of dominants through disease or outbreaks of specialist herbivores are not uncommon. Overall, these results highlight the prevalent contribution of high performing species to ecosystem functions and challenge the notion that subordinate and rare species can serve as reliable insurance. This idea was further confirmed in a recent meta‐analysis conducted by Avolio et al. (2019), which reported that most studies found that all measures of ecosystem function were diminished when a dominant species was removed.

One could argue that a complete loss of a dominant species might be an extreme event, and that subordinate and rare species can still provide some level of redundancy by compensating for the natural interannual fluctuations of a dominant. However, a recent study conducted on the same experimental site demonstrated that only two additional subordinate species were enough to compensate for the natural interannual fluctuations in the abundance of *M. caerulea* to the same level as in highly diverse communities (Lisner et al., 2024). This is in agreement with Munson and Lauenroth (2009), who showed that only subdominants increase in abundance after the removal of a dominant, further questioning the need for a high level of diversity as a source of redundancy.

It is worth noting that contrasted results were found in the belowground compartment of the vegetation, where the loss of *M. caerulea* neither affected the total standing root biomass nor the seasonal root growth. Hence, subordinate species were able to maintain a high belowground phytomass, which constitutes an important C stock in grassland ecosystems. Hernández et al. (2022) also demonstrated that belowground phytomass was less responsive to the absence of dominants, but studies investigating the contribution of low‐abundance species to belowground phytomass are generally rare. The mechanisms by which the remaining species compensate for the dominant loss remain unclear and represent an exciting avenue for future research. For example, like for the aboveground phytomass, we can speculate that an increase in the contribution of a few subdominant species and no change in the contribution of rare species (constituting most of the diversity) might be enough to maintain the C stock in the belowground phytomass. Only a detailed identification of the belowground species composition, using molecular analyses, for example, would allow us to investigate these responses.

### Biodiversity as a source of complementarity?

Subordinate and rare species can also contribute significantly to ecosystem functions through a complementarity effect (Mariotte, 2014). Indeed, low‐abundance species often constitute most of the trait diversity in local communities (Díaz et al., 2003) and rare species tend to display rare trait combinations (Leitão et al., 2016; Mouillot et al., 2013). Therefore, it has been hypothesized that the trait diversity provided by subordinate and rare species can be associated with niche complementarity and, more importantly, that the aggregated effect of these species can enhance ecosystem functions to a higher level than dominants alone (Lyons et al., 2005; Lyons & Schwartz, 2001). Although we did not measure the trait diversity formed by subordinate and rare species, we directly quantified the functional consequences of their removal on several ecosystem functions and across seasons. We found that, during the main vegetative season, in late spring and summer, monocultures of a dominant were able to maintain all measures of ecosystem function (above and belowground) at the same level as species‐rich controls (see also Lisner et al., 2023). Nevertheless, both aboveground and belowground ecosystem functions (see next section for soil functions) were depleted by the removal of subordinate and rare species in early spring and autumn, likely reducing the annual C input at the ecosystem level. Thus, plant diversity served as complementary support to enhance ecosystem functions outside the dominant‐driven peak of vegetation. Interestingly, we observed consistent trends along the diversity gradient rather than a sudden loss of function at low diversity levels (see Figure 4), supporting the idea of an aggregated effect of biodiversity with no quick saturation. This result may be explained by complementarity in species phenology, with subordinate species active earlier and later than the growth period of *M. caerulea*. This temporal niche differentiation between species has been demonstrated in the same study site through successive harvests of aboveground phytomass and species sorting (Doležal et al., 2019). This study also revealed that phenological complementarity was associated with differentiation in resource‐conservative and resource‐acquisitive life strategies between subordinate and dominant species, respectively. Similarly, it has been shown that subordinate species generally outperform dominants in resource use efficiency, particularly in the early and late seasons (Fitter, 1986; Pornon et al., 2007; Theodose et al., 1996). Associated with their shifted phenology, this high resource use efficiency allows low‐abundance species to be maintained in the community by escaping strong competition from dominants.

Overall, we found no evidence for a complementarity effect of biodiversity at the peak of the vegetation when dominants can maintain, alone, the same level of ecosystem functions as species‐rich communities. These results contrast with that of traditional BEF experiments showing that complementarity plays an important role at the peak of the vegetation in artificial communities (Cardinale et al., 2007; Ravenek et al., 2014) and tend to support the alternative hypothesis (the mass‐ratio hypothesis: Grime, 1998) for natural communities (see also Smith et al., 2020; Smith & Knapp, 2003). However, we found a consistent effect of an increasing diversity loss on both aboveground and belowground compartments of the vegetation in the early and late seasons, probably due to the loss of species with complementary phenological and resource‐acquisitive strategies. Whether the same conclusions apply to other systems remains to be tested, especially in communities dominated by species with a longer growing season than *M. caerulea*. Surprisingly, this temporal aspect of the complementarity effect of biodiversity has been rarely considered in BEF experiments, as most studies usually collect data at the peak of the vegetation only (but see Stevens & Carson, 2001). It suggests that the role of real‐world biodiversity in ecosystem functions may have been underestimated so far, especially outside of the peak season.

### Plant diversity and soil functions

Plant diversity is expected to be a key driver of soil organic C formation and storage (Bai & Cotrufo, 2022) through higher belowground C inputs (Lange et al., 2015) and by promoting microbial growth, turnover, and necromass (Prommer et al., 2020). Most of these results have been confirmed in artificial BEF experiments (references hereabove and also Fornara & Tilman, 2008; Lange et al., 2023; Steinbeiss et al., 2008). Other studies demonstrated that the restoration of grassland biodiversity enhanced soil organic C content (De Deyn et al., 2011; Klopf et al., 2017; Yang et al., 2019) and global analyses tend to confirm the relationship between plant diversity and soil organic C content (Chen et al., 2018, 2019), especially in warm and arid climates (Spohn et al., 2023). Mariotte et al. (2013) even demonstrate significant effects of subordinate removal on the soil microbial composition, soil respiration, and litter decomposition. Surprisingly, none of the treatments of species removal affected soil functions in our study. Despite strong effects on the aboveground phytomass and litter production (especially in the experiment where *M. caerulea* was removed for more than 25 years), soil contents of total and dissolved organic C remained unchanged. Similarly, we found no effect of species loss on the soil microbial activity (using seasonal measurement of soil basal respiration and decomposition rate of two types of standardized litter). One could argue that the small size of our experimental plots can explain this discrepancy. However, a previous study conducted on the same experimental site and with the same plot design demonstrated the strong effects of fertilization and cessation of mowing on soil functions (Kotas et al., 2017) and confirmed the lack of response due to the removal of *M. caerulea*.

Our results suggest that changes in aboveground litter production found in both experiments had little influence on the soil organic content. The preservation of soil functions may be due to the lack of response to species loss of the standing belowground phytomass, which constitutes a large proportion of the phytomass in grasslands. The latter might be the main driver of belowground C input through root exudates and belowground litter production. We further demonstrated that the loss of subordinate and rare species in the SubRarRem experiment did not affect the vertical distribution of roots (except a small decrease in the top 5 cm with species loss) nor their traits, challenging the idea of a strong belowground niche complementarity and suggesting little trait differentiation between species (Gracia et al., 2025). A better understanding of the mechanisms in play would require investigating the contribution of individual species to the belowground phytomass and the consequences for root exudates and belowground litter production and quality. Finally, a better characterization of the microbial communities and their capacity to decompose belowground litter could help us decipher the complex interactions between C input and outputs in the soil (e.g., Wagg et al., 2014).

## CONCLUSIONS

Our study underscores the critical importance of dominant species in driving ecosystem functioning at the peak of the vegetation, challenging traditional assumptions regarding the redundancy provided by subordinate species. While we observed complementarity effects manifesting outside the peak vegetation period, particularly during early spring and autumn, the absence of a significant impact on soil functions suggests more intricate interactions at play. This highlights the necessity of comprehensive seasonal measurements to accurately discern the effects of biodiversity on ecosystem dynamics. Testing whether similar conclusions apply to other ecosystems and for ecosystem functions beyond carbon dynamics is an exciting perspective for future research.

Overall, our study highlights the complex interplay between biodiversity and ecosystem functions, emphasizing the need for a nuanced understanding of how biodiversity loss affects ecosystem processes in real‐world ecosystems. Further research into the mechanisms driving the contributions of low‐abundance species to ecosystem functions, both aboveground and belowground, is essential for informing effective conservation and management strategies in the face of ongoing biodiversity decline.

## AUTHOR CONTRIBUTIONS

Aleš Lisner and Jan Lepš conceived the long‐term experiments. Jules Segrestin conceived the ideas for the study. Tomáš Hájek, Veronika Jílková, Jan Lepš, Aleš Lisner, and Jules Segrestin designed the methodology. Eva Janíková, Jan Lepš, Aleš Lisner, Marie Konečná, Jules Segrestin, and Tereza Švancárová conducted the experiment. Eva Janíková, Aleš Lisner, Marie Konečná, Jules Segrestin, and Tereza Švancárová collected the data. Jules Segrestin analyzed the data. Jules Segrestin led the writing of the manuscript. All authors contributed critically to the drafts and gave final approval for publication.

## CONFLICT OF INTEREST STATEMENT

The authors declare no conflicts of interest.

## Supporting information


Appendix S1.

## Data Availability

Data (Segrestin et al., 2025) are available in Zenodo at https://doi.org/10.5281/zenodo.14967638.
