# Supplementary material for: Biodiversity loss disrupts seasonal carbon dynamics in a species‐rich temperate grassland
Source: Ecology. 2025 May 8;106(5):e70091. doi: 10.1002/ecy.70091 (PMC12060612; doi:10.1002/ecy.70091)
Supplement: Supplementary file 1 — Appendix S1. [file ECY-106-e70091-s001.pdf]

APPENDIX S1

Article title: Biodiversity loss disrupts seasonal carbon dynamics in a species-rich temperate grassland

Journal: Ecology

Authors: Jules Segrestin, Aleš Lisner, Lars Götzenberger, Tomáš Hájek, Eva Janíková, Veronika Jílková, Marie Konečná, Tereza Švancárová, and Jan Lepš

Table S1. Post-hoc Tukey test on the linear mixed-effect model testing the effect of the removal treatment on the litter biomass in SubRarRem. Significant differences (P values < 0.05) are shown in bold.

	Estimate	Std. Error	z value	P value
12 Dom - Control	0.04	0.096	0.43	0.993
6 Dom - Control	0.22	0.096	2.27	0.156
3 Dom - Control	0.18	0.096	1.88	0.327
1 Dom - Control	0.31	0.096	3.19	**0.012**
6 Dom - 12 Dom	0.18	0.096	1.83	0.354
3 Dom - 12 Dom	0.14	0.096	1.45	0.595
1 Dom - 12 Dom	0.27	0.096	2.75	**0.046**
3 Dom - 6 Dom	-0.04	0.096	-0.38	0.995
1 Dom - 6 Dom	0.09	0.096	0.92	0.889
1 Dom - 3 Dom	0.13	0.096	1.30	0.689

Table S2. Wald chi-square tests on linear mixed-effect models akin to a repeated measures ANOVAs, including the sampling date, removal treatment, and their interaction as fixed effects. The models also included the plot and block identities as random factors. DomRem is the experiment where the dominant species (*Molinia caerulea*) was removed from the vegetation and SubRarRem is the experiment where 5 levels of subordinate and rare species removal were conducted. Significant effects (P values < 0.05) are shown in bold.

	DomRem			SubRarRem		
	χ^2	df	P value	χ^2	df	P value
**NEE**						
Time	1672.3	11	**< 0.0001**	4546.1	10	**< 0.0001**
Removal	2.6	1	0.105	12.3	4	**0.015**
Time x Removal	62	11	**< 0.0001**	46.9	40	0.211
**Vegetative height**						
Time	2230.3	25	**< 0.0001**	4569.7	25	**< 0.0001**
Removal	48.3	1	**< 0.0001**	6.03	4	0.197
Time x Removal	297.5	25	**< 0.0001**	130.5	100	**0.022**
**Root growth**						
Time	136.5	3	**< 0.0001**	264	3	**< 0.0001**
Removal	1.3	1	0.254	14.1	4	**0.007**
Time x Removal	2	3	0.570	11.8	12	0.461
**Basal soil respiration**						
Time	14.2	3	**0.003**	47.3	3	**< 0.0001**
Removal	0.2	1	0.698	5.9	4	0.207
Time x Removal	2.1	3	0.546	20.2	12	0.062
**Litter mass loss (green)**						
Time	205.9	3	**< 0.0001**	148.8	3	**< 0.0001**
Removal	0.2	1	0.639	1.3	4	0.864
Time x Removal	0.5	3	0.921	17.8	12	0.123
**Litter mass loss (rooibos)**						
Time	124.8	3	**< 0.0001**	214.2	3	**< 0.0001**
Removal	1.3	1	0.249	4.4	4	0.349
Time x Removal	1.6	3	0.661	17.3	12	0.139

Table S3. Effect of species removal on net ecosystem CO_2 exchange. Wald chi-square tests on separated linear mixed-effect models for each sampling date (DOY: day of the year). The models also included the plot and block identities as random factors. DomRem is the experiment where the dominant species (*Molinia caerulea*) was removed from the vegetation and SubRarRem is the experiment where 5 levels of subordinate and rare species removal were conducted. Significant effects (P values < 0.05) are shown in bold. These multiple individual tests should be interpreted carefully due to the temporal autocorrelation between successive sampling and the increasing probability of type I error with multiple testing.

	**DomRem**			**SubRarRem**		
**DOY**	χ^2	**df**	**P value**	χ^2	**df**	**P value**
55	7.1	1	**0.008**	0.5	1	0.477
82	36.7	1	**< 0.0001**	0.5	1	0.471
119	6.7	1	**0.010**	1.7	1	0.195
139	4.4	1	**0.037**	20.4	1	**< 0.0001**
159	4.4	1	**0.037**			
165	0.4	1	0.535	0.01	1	0.899
172	0.2	1	0.634	11.0	1	**0.001**
200	48.9	1	**< 0.0001**	1.0	1	0.320
228	5.3	1	**0.022**	2.0	1	0.153
249	2.1	1	0.148	7.6	1	**0.006**
278	0.3	1	0.573	4.7	1	**0.030**
318	15.8	1	**< 0.0001**	8.3	1	**0.004**

Table S4. Effect of species removal on vegetative plant height. Wald chi-square tests on separated linear mixed-effect models for each sampling date (DOY: day of the year). The models also included the plot and block identities as random factors. DomRem is the experiment where the dominant species (*Molinia caerulea*) was removed from the vegetation and SubRarRem is the experiment where 5 levels of subordinate and rare species removal were conducted. Significant effects (P values < 0.05) are shown in bold. These multiple individual tests should be interpreted carefully due to the temporal autocorrelation between successive sampling and the increasing probability of type I error with multiple testing.

	**DomRem**			**SubRarRem**		
**DOY**	χ^2	**df**	**P value**	χ^2	**df**	**P value**
55	0.2	1	0.637	2.8	1	0.096
82	0.2	1	0.665	0.2	1	0.632
111	1.7	1	0.197	2.7	1	0.098
119	0.1	1	0.730	0.4	1	0.523
124	0.2	1	0.627	0.0	1	0.920
130	0.0	1	0.901	1.1	1	0.292
139	2.2	1	0.137	0.0	1	0.839
146	3.0	1	0.081	2.6	1	0.109
154	8.5	1	**0.004**	0.0	1	0.897
159	28.6	1	**< 0.0001**	3.1	1	0.079
164	103.6	1	**< 0.0001**			
171				2.4	1	0.119
172	25.3	1	**< 0.0001**	0.1	1	0.724
179	30.7	1	**< 0.0001**	0.0	1	0.992
188	20.7	1	**< 0.0001**	3.2	1	0.075
200	40.8	1	**< 0.0001**	4.4	1	**0.035**
209	14.1	1	**< 0.001**	7.1	1	**0.008**
221	29.6	1	**< 0.0001**	14.8	1	**< 0.001**
228	130.5	1	**< 0.0001**	10.4	1	**0.001**
241	13.8	1	**< 0.0001**	13.7	1	**< 0.001**
249	70.2	1	**< 0.0001**	5.8	1	**0.016**
272	90.5	1	**< 0.0001**	6.8	1	**0.009**
278	17.1	1	**< 0.0001**	3.0	1	0.084
292	65.3	1	**< 0.0001**	0.6	1	0.443
304	21.3	1	**< 0.0001**	1.9	1	0.165
318	0.8	1	0.373	4.0	1	**0.046**
339	1.2	1	0.282	3.0	1	0.084

Table S5. Effect of subordinate and rare species removal on the seasonal production of belowground phytomass. Wald chi-square tests on separated linear mixed-effect models for each season. The models also included the plot and block identities as random factors. Significant effects (P values < 0.05) are shown in bold.

**Season**	χ^2	**df**	**P value**
Spring	7.7	1	**0.006**
Summer	1.6	1	0.207
Autumn	17.8	1	**< 0.0001**
Winter	0.7	1	0.414

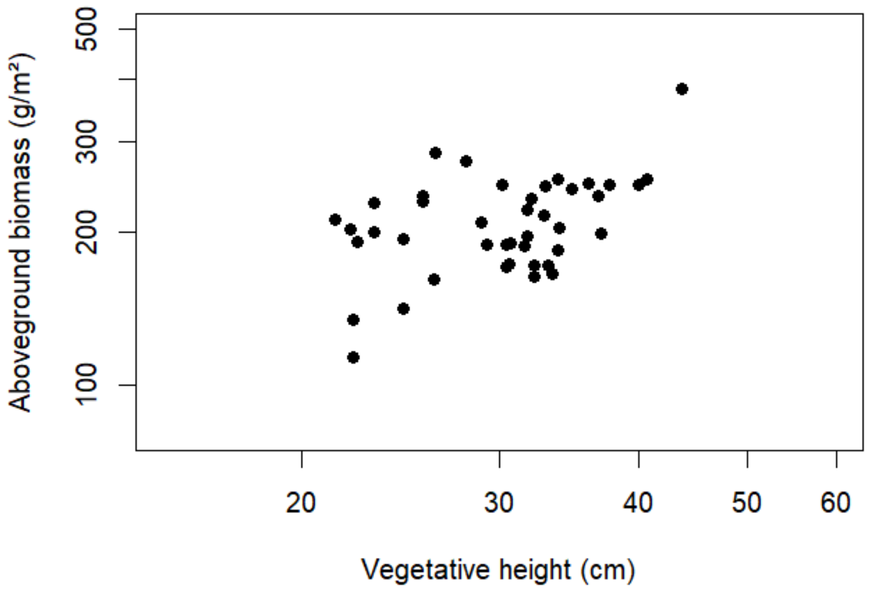

Figure S1. The correlation between vegetation height and the aboveground phytomass at the peak of vegetation is significant (Pearson correlation test, $r = 0.45$, $t = 3.2$, $df = 40$, $P = 0.002$). The fact that the relationship is significant despite the lack of differences between treatments in SubRarRem (all treatments showed similar values in biomass and height) demonstrates that plant height is a good marker of aboveground phytomass.
